# Brazilian Organic Honeydew Reduces In Vitro and In Vivo Periodontal Disease-Related Subgingival Biofilm †

**DOI:** 10.3390/foods14060997

**Published:** 2025-03-14

**Authors:** Diego Romário-Silva, Marcelo Franchin, Bruno Bueno-Silva, Ana Sofia Martelli Chaib Saliba, Janaína Orlandi Sardi, Thayna Alves-Ferreira, Josy Goldoni Lazarini, Gustavo Aparecido Cunha, Severino Matias de Alencar, Pedro Luiz Rosalen

**Affiliations:** 1 Department of Biosciences, Piracicaba Dental School, University of Campinas (UNICAMP), Piracicaba 13414-903, SP, Brazilpedro.rosalen@unifal-mg.edu.br (P.L.R.); 2 Graduate Program in Integrated Dental Sciences, School of Dentistry of the University of Cuiabá, Cuiabá 78065-900, MT, Brazil; odontologia@fasipecuiaba.com.br; 3 Graduate Program in Biological Sciences, Federal University of Alfenas (Unifal-MG), Alfenas 37130-001, MG, Brazil; 4 Dental Research Division, Guarulhos University, Guarulhos 07023-070, SP, Brazil; janaina.cassia@prof.ung.br; 5 Department of Agri-Food Industry, Food and Nutrition, “Luiz de Queiroz” College of Agriculture, University of São Paulo (USP), Piracicaba 13418-900, SP, Brazil; sofiasaliba@usp.br (A.S.M.C.S.); smalencar@usp.br (S.M.d.A.); 6 Graduate Program in Pharmaceutical Sciences, Federal University of Alfenas (Unifal-MG), Alfenas 37130-001, MG, Brazil; gustavo.cunha@sou.unifal-mg.edu.br

**Keywords:** honeydew, functional food, periodontal disease, antibiofilm activity, chemical characterization

## Abstract

We investigated the antimicrobial properties and effects on bone resorption of Brazilian organic honeydew (OHD) from the Bracatinga tree (*Mimosa scabrella* Benth.), a rare honey certified with Denomination of Origin, using a periodontal disease model. Antibiofilm activity was assessed using a subgingival biofilm adhered to the Calgary device. Biofilms were treated with OHD, chlorhexidine (0.12%), or a vehicle twice daily for 1 min starting on day 3, at concentrations of 2× and 10× the minimum inhibitory concentration (MIC). We employed a ligature-induced chronic periodontal disease model and challenged it with *Porphyromonas gingivalis* in C57BL/6 mice. The chemical profile of OHD was analyzed using LC-ESI-IT-MS/MS. Results were evaluated by measuring bone loss and microbial composition of the ligature biofilm through DNA–DNA hybridization. OHD demonstrated significant activity against *P. gingivalis* (MIC 4%, MBC 6%) and reduced biofilm viability by 80% in vitro. In vivo, OHD decreased microbial populations and decreased bone loss associated with periodontal disease. Chemical analysis identified seven compounds in OHD, including five flavonoids and two lignans. This Brazilian honeydew from the Atlantic Forest exhibits strong antimicrobial properties and potential as a functional food for oral health, offering a promising alternative for the control and prevention of periodontal disease.

## 1. Introduction

Natural products have been used since ancient times to treat human diseases, and the popular knowledge in traditional medicine provides information for the discovery of novel medicines and therapies from products of animal, plant, and mineral origin [1]. Honey is a food produced by bees, especially by *Apis mellifera*, that is extensively used due to its several biological activities [2]. Therefore, this food was used to treat wounds and tissue lesions and was a common medical practice in hospitals across Europe until the 1970s, due to its antimicrobial and anti-inflammatory activities, stimulation of tissue repair, deodorant action, and injury tissue debris [3]. Currently, modern medicine has rediscovered the honey therapeutic properties in infection treatments in hospitalized patients, as an adjuvant and alternative therapy against resistant microorganisms [4,5].

The growing interest in an atypical honey variety, commonly referred to as honeydew honey, has stemmed from its distinct nutritional, sensory, and potential therapeutic attributes [6,7]. Honeydew honey, derived by bees (*Apis mellifera*) from secretions of living plant parts or excretions of plant-sucking insects, stands in contrast to the more familiar blossom honey produced from flower nectar [8]. Despite its higher richness in phytochemical components and the consumption of honeydew honey, the literature is still limited regarding its biological properties when compared to floral honeys. Additionally, the chemical characterization of honeydew honey remains challenging due to its complex composition and the variability in its botanical and entomological origins [9,10].

The microorganisms’ resistance to antibiotics has increased and is considered a public health problem [11]. The pharmaceutical industry invests in the research and development of novel medicines with an alternative mechanism of action; however, microorganisms increasingly possess an arsenal to overcome all obstacles to medicine. Moreover, the search for alternative therapies, and sources for the bioprospection of novel medicines, must be stimulated [12]. Several studies have shown floral honey as a promising antimicrobial in different microorganisms causing human diseases, such as protozoa [13,14], fungi [15], and bacteria [16].

Recent studies have investigated honeydew honey as a functional food with potential health benefits [7,17], although there remains a lack of detailed information regarding its specific indications. Research indicates that honeydew honey possesses antioxidant, antimicrobial, and anti-inflammatory activities, attributed to bioactive compounds such as phenolics, proteins, and amino acids [6,18,19,20,21]. This honey has a higher concentration of phenolic compounds compared to flower honeys, resulting in potent antioxidant activity [22,23]. Furthermore, its anti-inflammatory property has been highlighted, demonstrating efficacy in the treatment of ulcers and wounds [24].

Regarding antimicrobial activity, honeydew honey shows superior performance, particularly against *Staphylococcus aureus* and *Pseudomonas aeruginosa*, due to its unique composition and higher acidity [25,26,27]. However, few studies have demonstrated its antimicrobial activity against bacteria responsible for human infections [6,28], and none against periodontal pathogens. A search in the PubMed© database was conducted in March 2025, with no date restrictions, revealing only 64 indexed articles for the term ‘Antimicrobial honeydew’, while the term ‘Antimicrobial honey’ (using the Boolean operator ‘not’ to exclude ‘honeydew’) resulted in 2810 indexed articles. Despite the various biological properties reported for the honeydew variety, studies on its antimicrobial activity remain limited when compared to floral honey. Therefore, further research into this variety is essential, particularly considering the current global issue of multi-resistant infections.

The microorganisms present in the oral microbiota can cause different types of diseases. Periodontal disease is one of the most prevalent pathologies in the oral cavity and similar to dental caries, both are biofilm-dependent [29,30]. The consequences of periodontal disease include tooth loss, inflammation, halitosis, and difficulty chewing. The supragingival and subgingival biofilm formation is a crucial factor to establish and subsequently facilitate the progress of periodontal disease [31]. The microbial colonization by the periodontopathogens and the inflammatory process are responsible for the clinical damage caused by periodontal disease [32].

The orientation of prevention against periodontal disease includes a toothbrush, dental floss, mouthwash, and frequent visits to the dentist [33]. Nonetheless, until now, there has been no report of using functional foods as an alternative therapy to prevent or treat periodontal disease. Despite honey’s antimicrobial and anti-inflammatory properties having already been related in the scientific literature, the research of these biological activities regarding periodontal disease is incipient primarily about the antimicrobial activities against oral biofilms, especially related to honeydew honey produced organically. Therefore, we evaluated in vitro and in vivo the effect of Brazilian organic honeydew (OHD) on microbial components and bone resorption in the periodontal disease model.

## 2. Materials and Methods

### 2.1. Organic Honey Georeferencing, Collection, and Extraction

A sample Brazilian organic honeydew (OHD) from the Bracatinga tree (*Mimosa scabrella* Benth.) was collected and geographically referenced in a preserved Atlantic rainforest region from South Paraná State, in the city of General Carneiro, Brazil (S 26°25′, W 51°19′) between the months of December 2015 and February 2016. The collection was carried out under the permission of the Brazilian Ministry of Environment (Council for the Administration and Management of Genetic Heritage–CGEN/CNPq, # AE0DBB2). The honeydew honey samples were stored in airtight containers at room temperature (20–25 °C), protected from light and humidity, to preserve their stability and properties. Prior to analysis, the honeydew samples were diluted at several concentrations using a saline or culture medium and sterilized by filtration.

### 2.2. Polyphenol Honey Extraction

The polyphenol fraction was extracted using a previously described method [34], with modifications. OHD weighing 60 g was separated and dissolved in 300 mL of water. Later, the pH of the solution was adjusted at pH 2 and then mixed with 90 g of AmberliteVR XAD-2 resin (pore size 9 nm; particle size 0.3–1.2 nm; Sigma-Aldrich, St. Louis, MO, USA) for 15 min using a magnetic stirrer. Then, the mixture was packed in a glass column (40 cm, 2 cm). The solution in the column was then eluted with 500 mL of acid water (pH 2.0) and then with 500 mL of neutral distilled water to remove the sugars. The phenolic compounds adsorbed on the solid phase were eluted with 500 mL of methanol and then concentrated on a rotary evaporator (Rotavapor® R-151, Büchi Labortechnik AG, Flawil, Switzerland) under low pressure at 50 °C. Finally, all the OHD extracts (OHD-Es) obtained were lyophilized (model K108, Liotop, São Carlos-SP, Brazil) and stored at 22 °C until analyzed.

### 2.3. Microbial Susceptibility

A *Porphyromonas gingivalis* W83 strain was used as a periodontal pathogen. The OHD was diluted in a BHI-plus-TSB culture medium and yeast supplemented with hemin and menadione. Metronidazole, a monodrug, was used as a positive control. The final concentration in the wells was from 60% to 10% (*w*/*v*). The microorganism susceptibility was carried out by the microdilution technique, and the same technique was used to determine the minimum inhibitory concentration (MIC) and Minimum Bactericidal Concentration (MBC). These assays were conducted in 96-well microplates [35].

### 2.4. Evaluation of Antibiofilm Activity

#### 2.4.1. In Vitro Model of Subgingival Biofilm Multispecies

The in vitro model of subgingival multispecies biofilm was conducted as previously described [36]. All bacterial strains included in the multispecies biofilms are listed in Table S1 above. All microorganisms were grown in a specific solid culture medium and appropriate conditions for each strain. After 24 h, the bacterial strain was mixed with BHI (Becton Dickinson, Sparks, MD, USA) supplemented with 1% hemin for 24 h. The single microorganisms’ suspensions at 10^8^ CFU/mL of cells (standardized by spectrophotometer) were diluted, and 100 µL of the inoculum containing 10^6^ cells was mixed with 11.8 mL of BHI broth supplemented with 1% hemin and 5% of sheep blood. The biofilm inoculum consisted of 15 mL with 10^6^ cells of each bacterial strain. The multispecies biofilm formation was induced in a Calgary biofilm device (CBD) on a 96-well microplate (Nunc; Thermo Scientific, Roskilde, Denmark). Aliquots of 150 µL of each inoculum were added to the wells, which corresponded to 10^4^ cells of each bacterial strain. A top with polystyrene pins was used to seal the 96-well microplate (Nunc TSP system; Thermo Scientific, Roskilde, Denmark). The microplates were incubated at 37 °C under anaerobic conditions. After 3 days, the medium consumed by the bacteria was replaced, and the biofilms were maintained at 37 °C in anaerobic conditions for four days to obtain mature biofilms of seven days.

#### 2.4.2. OHD Treatment on Biofilm

After these 3 days, the mature biofilm received organic honeydew twice a day for more than 4 consecutive days. The biofilm-coated pins were transferred to 96-well microplates containing diluted honey at 2× and 10× CIM, the dilution vehicle (negative control), and 0.12% chlorhexidine (Periogard, Colgate, positive control). The mature biofilm remained in contact with the treatments for 1 min, twice a day. After this procedure, the biofilm-coated pins were washed with PBS and returned to the same culture medium. The three independent experiments were carried out in triplicate.

#### 2.4.3. Metabolic Biofilm Activity and DNA–DNA Hybridization (Checkerboard Assay)

The metabolic activity of multispecies biofilms treated with honey, extracts, and controls was measured using 2,3,5-triphenyltetrazolium chloride (TTC; catalog number 17779, Fluka Analytical, Darmstadt, Germany) in a spectrophotometer. To measure the metabolic activity of biofilms, the coated pins were transferred to 96-well microplates with 200 µL of a brain heart infusion (BHI) medium supplemented with 1% hemin and 10% of TTC solution (1%). The microplates were incubated in anaerobic conditions for 24 h at 37 °C. The reduction of TTC to red formazan was read at 485 nm using a spectrophotometer. Three biofilm-coated pins from each group were added in tubes, washed in Phosphate-Buffered Saline (PBS), and mixed with 100 µL of Tris-ethylenediaminetetraacetic acid (Tris-EDTA) buffer [10 mM Tris-hydrochloride (Tris-HCl), 1 mM EDTA (pH 7.6)], followed by the addition of 100 µL of sodium hydroxide (NaOH) at 0.5 M. The above solution was sonicated for 10 min, and then the solution was neutralized with 0.8 mL of ammonium hydroxide (NH_4_OH) at 5 M. The samples were individually analyzed in terms of the presence and quantity of 34 species of bacteria, using the DNA–DNA hybridization technique [36].

### 2.5. Evaluation of OHD Potential in Periodontal Disease Induced by Ligature and P. gingivalis in Mice

#### 2.5.1. Animals

Male SPF (specific-pathogen free) C57BL/6JUnib mice, purchased from CEMIB/UNICAMP (Multidisciplinary Center for Biological Research, SP, Brazil), weighing 22–25 g. All animals were housed in a vivarium under 40–60% humidity and temperature (22 ± 2 °C) control with a 12 h light–dark cycle, with access to food and water ad libitum. For the experiment, all animals were deprived of food for 8 h before oral administration.

This study complied with the National Council for Animal Experimentation Control guidelines for the care and use of animals in scientific experimentation. All animals were euthanized by deepening anesthesia with ketamine (300 mg/kg) and xylazine (30 mg/kg), followed by cervical dislocation. The study protocol was previously approved by the Institutional Ethics Committee on Animal Research at the University of Campinas (CEUA/UNICAMP, Protocol Number 5348-1).

#### 2.5.2. Periodontal Disease Inducted by Ligature Model Plus *P. gingivalis* W83

A *P. gingivalis* W83 strain was cultivated in a brain heart infusion (BHI, BD-Difco, Sparks, MD, USA) medium, supplemented with 1 mg/mL of hemin, and 0.5 mg/mL of menadione, and incubated in anaerobiosis at 37 °C for two days. To induce bone loss, a ligature 5–0 silk suture thread (Roboz Surgical Instrument Co., Gaithersburg, MD, USA) was previously contaminated with *P. gingivalis* (10^7^ CFU/mL diluted in carboxymethylcellulose and PBS). This ligature-induced periodontitis was tied around the left upper second molar in mice. The ligature was gently tied avoiding damage to the periodontal tissue [37]. Subsequently, mice were inoculated with *P. gingivalis* (100 µL–10^7^ CFU/mL diluted in carboxymethylcellulose and PBS) 3 times a day until day 4, counting from the beginning of the treatment.

#### 2.5.3. Organic Honeydew (OHD) Treatment

The experimental design was conducted as follows: the experimental group was divided in groups of 6 mice: Group 1 (no ligature and treated with NaCl); Group 2 (with ligature and treated with NaCl); Group 3 (with ligature and treated with 100 µL of OHD 40% (*w*/*v*), orally 5×/day); and Group 4 (with ligature and treated with 100 µL of OHD in natura 5×/day). All mice were treated until day 7 and were subsequently euthanized.

#### 2.5.4. Bone Loss Measure

Mice jaws were supplemented with 3% hydrogen peroxide and mechanically dissected. To distinguish the cement–enamel junction, the jaws were stained with 3% methylene blue. The face-palatal junctions were photographed with a 2.5× extension using a stereoscopic microscope (ZEISS CL1500 ECO) and a digital camera (Canon E0S 1000D). The images were obtained and analyzed by the software Image J (Bethesda, MD, USA, version 1.54j). For quantitative analysis, the distomesial area (mm^2^) between the cementoenamel junction and the alveolar bone crest on the palatal side of the first and second molars was measured, as previously described [38].

#### 2.5.5. Evaluation of the Tooth Caries Presence

The tooth caries decay and its severity were quantified on smooth and grooved surfaces (Ds, exposed dentin; Dm, 3/4 of the affected dentin; Dx, all affected dentin). The determination of the caries score was performed by two calibrated examiners [39].

### 2.6. Chemical Profile by LC-ESI-IT-MS/MS

The OHD obtained in Section 2.2 was resuspended in distilled water at a 10 mg/mL concentration and filtered in 0.22 µm Regenerated Cellulose Syringe Filters prior to analysis. An HPLC system coupled to an SPD-20A UV–vis detector (Shimadzu Co., Tokyo, Japan) and to an Amazon Speed ETD mass spectrometer (Bruker Daltonics Corporation, Billerica, MA, USA) with an electrospray ionization (ESI) source operating in negative mode was used to analyze the organic honey sample, as previously described [40]. The nebulizer was at 27 psi and the dry gas at 7 L/min. Separation was performed on a Phenomenex Luna C18 column (250 × 4.6 mm, 5 μm) at 30 °C. The mobile phase consisted of water–formic acid (99.75%:0.25%, *v*/*v*) (A) and acetonitrile–water–formic acid (59.75%:40%:0.25%, *v*/*v*) (B), and the solvent flow rate was 1.0 mL/min. Gradient elution started with 10% B, and increased to 20% B (10 min), 30% B (20 min), 50% B (32 min), 90% B (40 min) and then decreased to 10% B (45 min). The data analysis was performed using the MAXIS 3G software (Bruker Daltonics, version 4.3).

### 2.7. Statistical Analysis

The data were checked for normality and submitted to a one-way analysis of variance (ANOVA) followed by Tukey’s post-hoc test. The results shown as not normal were submitted to a Kruskal–Wallis test followed by Dunnett’s post-hoc test. The results are expressed as mean ± standard deviation (SD). The results were considered significant at *p* < 0.05 and α = 5%.

## 3. Results

### 3.1. In Vitro Assays

OHD showed antimicrobial activity against *P. gingivalis* with MIC and MBC values of 4% and 7%, respectively (Table 1). Metronidazole, a gold standard effective against anaerobic microorganisms, was used as an internal positive control showing MIC and MBC values ranging from 0.039 and 0.078 µg/mL, respectively.

**Figure 1:**
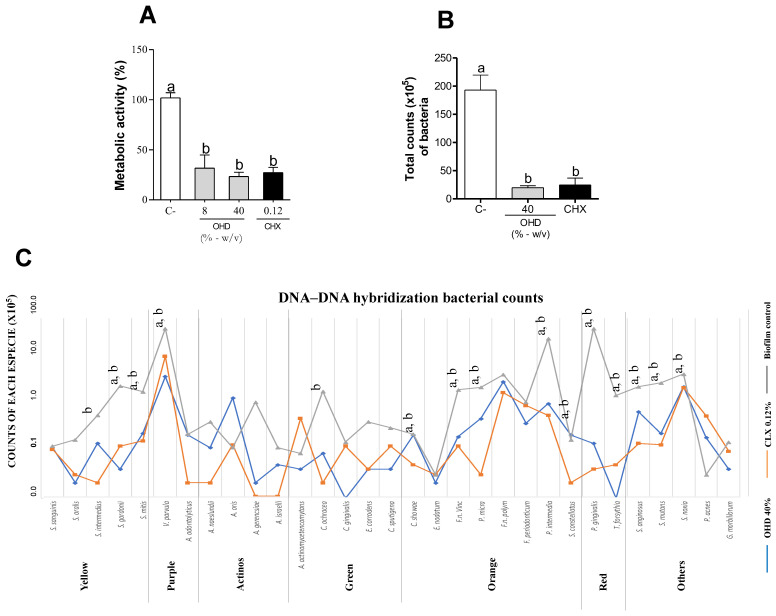
The antibiofilm activity of OHD (2×MIC 8%–10×MIC 40%) on subgingival multispecies biofilm (32 microorganisms) was assessed. Figure (**A**) illustrates the metabolic activity of the biofilm. Figure (**B**) shows the total bacterial count (10^5^) in biofilms treated with the negative control (vehicle; C-), 40% OHD, and 0.12% chlorhexidine. Results are expressed as mean ± SD from three separate assays (N = 9). Different letters indicate statistical significance. Figure (**C**) displays the mean count of each bacterial strain (10^5^) based on Socransky’s bacterial complexes. The bacterial complexes present in the ligature are labelled yellow, purple, actinomyces, green, orange, red, and others. The biofilm was treated with a vehicle, 40% OHD, and 0.12% chlorhexidine. The letter (a) indicates statistical significance between biofilms treated with OHD and those treated with the vehicle. The letter (b) indicates statistical significance between biofilms treated with chlorhexidine and those treated with the vehicle.

The antibiofilm results shown in Figure 1A indicate that OHD, at both 2× MIC and 10× MIC concentrations, reduced the metabolic activity of the subgingival multispecies biofilm in vitro, with no statistical difference between the concentrations. There was no significant difference in the metabolic activity of the biofilm between OHD and the positive control, chlorhexidine (CHX) (*p* > 0.05). The total biofilm cell count was also significantly reduced compared to the untreated control, both by OHD and chlorhexidine, with no statistical difference between the treatments (*p* < 0.05).

Since verifying the biofilm viability, DNA–DNA hybridization (checkerboard assay) was carried out. Figure 1B represents the mean count of each bacterial strain based on the DNA–DNA hybridization (checkerboard assay). The treatment with OHD (40%) decreased the total bacterial count in the biofilm by 89% (*p* < 0.05) and did not show a statistical difference compared to the positive control, chlorhexidine (*p* > 0.05). Additionally, the treatment with OHD (40%) decreased the levels of the following 13 bacterial species compared to the vehicle group: *Streptococcus gordonii*, *Streptococcus mitis*, *Veillonella parvula*, *Capnocytophaga showae*, *Fusobacterium nucleatum vincentii*, *Prevotella micros*, *Prevotella intermedia*, *Streptococcus constellatus*, *Porphyromonas gingivalis*, *Tannerella forsythia*, *Streptococcus anginosus*, *Streptococcus mutans*, and *Streptococcus noxia*. The chlorhexidine positive control group reduced the levels of the following 15 bacterial species: *Streptococcus intermedius*, *Streptococcus gordonii*, *Streptococcus mitis*, *Veillonella parvula*, *Capnocytophaga ochracea*, *Capnocytophaga showae*, *Fusobacterium nucleatum vincentii*, *Prevotella micros*, *Prevotella intermedia*, *Streptococcus constellatus*, *Porphyromonas gingivalis*, *Tannerella forsythia*, *Streptococcus anginosus*, *Streptococcus mutans*, and *Streptococcus noxia*. The bacterial species marked in red, such as *Porphyromonas gingivalis* and *Tannerella forsythia*, showed the lowest levels after the OHD treatments, as seen in Figure 1C.

### 3.2. In Vivo Assays

**Figure 2:**
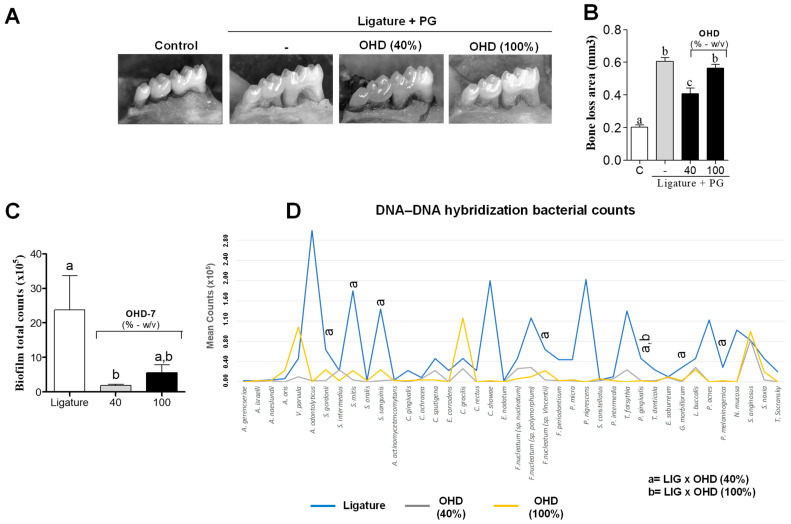
(**A**) Shows the hemimandibles of mice with ligature-induced periodontitis and *P. gingivalis* inoculation. The image includes a control group without disease and untreated (Control), a control group with disease and untreated (-), a group with disease treated with 40% OHD (OHD 40%), and a group with disease treated with raw OHD (OHD 100%). (**B**) Bone loss was assessed by measuring the general distance from the CEJ to the ABC in mice treated with the vehicle, 40% OHD, and raw OHD. Results are expressed as mean ± SD, and different letters indicate statistical significance (one-way ANOVA with Tukey’s post-hoc test, *p* < 0.05). (**C**) Total bacterial count (10^5^) in ligature-induced periodontitis in untreated mice (-), and mice treated with 40% OHD and raw OHD (100%). Results are expressed as mean ± SD, and different letters indicate statistical significance (Kruskal–Wallis test followed by Dunnett’s post-hoc test, *p* < 0.05). (**D**) Mean count of each bacterial strain (10^5^) present in the ligature, based on Socransky’s bacterial complexes, labelled yellow, purple, actinomyces, green, orange, red, and others. Data were analyzed by a Kruskal–Wallis test followed by Dunnett’s post-hoc test for each bacterial species separately. The letter (a) indicates statistical significance between the OHD 40%-treated group and the vehicle-treated group. The letter (b) indicates statistical significance between the raw-OHD-treated group and the vehicle-treated group.

Based on the in vitro results regarding antimicrobial activity and multispecies biofilm formation, we further investigated the in vivo activity of OHD in a periodontal disease model. Figure 2A illustrates the differences in the hemimandibles of mice with ligature-induced periodontitis inoculated with *P. gingivalis*. The periodontitis showed reduced bone resorption when orally treated with 40% OHD, as demonstrated in Figure 2B (*p* < 0.05). The oral administration of raw OHD did not show a statistical difference compared to the control group.

The total bacterial count, classified by Socransky complexes, was reduced after treatment with 40% OHD compared to the control, as shown in Figure 2B (*p* < 0.05). Figure 2C,D reveal a significant reduction in the total bacterial count of the red complex in both the 40% OHD-treated group and the raw-OHD-treated group (*p* < 0.05). None of the mice in any of the groups were affected by caries lesions and/or white-spot lesions.

### 3.3. Chemical Composition

Seven compounds were found in the OHD extract (as shown in Table 2) and tentatively identified using LC-ESI-IT-MS/MS. The results were compared to the exact mass and fragmentation data from the literature. Five of these compounds were found to belong to the flavonoid class, a group of polyphenols known for their high bioactivity, including anti-inflammatory properties. Apigenin (*m*/*z* 256.07), a 7-hydroxyflavone, was tentatively identified in the sample in the form of two isomers with retention times of 5.5 min (isomer 1) and 18.2 min (isomer 2).

Quercetin (*m*/*z* 303.05) was tentatively identified through the precursor ion *m*/*z* 284. Hesperidin (*m*/*z* 611.18), a flavanone glycoside, was formed by hesperitin (aglycone form) and sucrose (glycoside). It was tentatively identified through the precursor ion *m*/*z* 302, referring to the aglycone form (C_16_H_14_O_6_). The compound (*m*/*z* 303.02) was tentatively characterized as the isoflavone 5,6,7,3′,4′-pentahydroxyisoflavone through precursor ions *m*/*z* 285 and 257.

The compound *m*/*z* 345.16 was tentatively identified as anhydrosecoisolariciresinol, which is believed to be derived from the dehydration reaction of a secoisolariciresinol molecule. Matairesinol (*m*/*z* 359.14) was tentatively identified through the precursor ion *m*/*z* 341.

## 4. Discussion

The antibacterial properties of honey are well documented in the scientific literature [41]. Numerous studies have demonstrated its activity against significant systemic pathogens, including methicillin-resistant *Staphylococcus aureus* (MRSA) [42], *Escherichia coli*, *Proteus mirabilis*, *Shigella flexneri*, and *Staphylococcus epidermidis* [43]. However, there is a paucity of studies examining the antibacterial activity of honey against oral microorganisms, particularly periodontal pathogens [16].

In previous research, we conducted a bioassay-guided screening of eight samples of Brazilian organic honey from the Atlantic Forest against cariogenic oral microorganisms [16] and also against the *P. gingivalis* W83 strain (unpublished data). All tested samples, including both honey (floral honey and honeydew honey) and its extracts, exhibited antibacterial activity against *P. gingivalis*. The organic honeydew honey from *Mimosa scabrella* (OHD), sample 7, showed the best results and was selected for further biological assays. OHD exhibited MIC and MBC values of 4% and 6% (*w*/*v*), respectively, against *P. gingivalis*. These results suggest that organic honey may be a promising functional food against periodontal pathogens such as *P. gingivalis*, warranting further research into its components and their role in periodontal disease.

Previous studies evaluated the antimicrobial activity of Manuka honey against various *P. gingivalis* strains, including ATCC 33277, M5-1-2, MaRL, and J361-1, showing a minimum inhibitory concentration (MIC) of 2% for all strains [44]. Another study tested Manuka and clover honey against *P. gingivalis* ATCC 33277, finding MIC values of 12.5% for both types of honey [45]. Manuka honey from New Zealand is highly valued for its antimicrobial activity, which is not linked to peroxides and is often used in medical treatments due to its medicinal properties [46,47]. In contrast, OHD’s antimicrobial activity is primarily attributed to the peroxides produced by its enzymatic complex, as well as other components like phenolic compounds [16].

In this study, Brazilian organic honey demonstrated promising MIC values against *P. gingivalis*, comparable to those found for Manuka honey. Other studies have evaluated Egyptian honey [48], Manuka honey, and a multifloral honey against oral microorganisms, including *P. gingivalis*, with positive results. However, the use of the agar diffusion method in these studies complicated comparisons, as this method is not recommended for honey due to its high viscosity. This can lead to issues such as incorrect well volumes and inadequate diffusion of active components, resulting in unreliable data [49,50].

The antimicrobial activity of honey is well-established, but there is no consensus among scientists on the optimal methodology for evaluating it [50]. Antimicrobial activity is just one of the initial steps in bioassay-guided studies aimed at discovering new substances with potential antimicrobial properties [51]. However, with the rise in antimicrobial resistance and the severity of biofilm-associated diseases, assessing antibiofilm activity is essential for new antimicrobial therapy candidates [52]. Microorganisms in biofilm form are organized in terms of architecture and integrity, making them up to 1000 times more resistant [53].

Biofilms are dense microbial communities that grow on surfaces and are encased in high molecular weight polymers they secrete. As bacteria form biofilms, they adapt to environmental changes, altering their genetic expression patterns and becoming highly resistant to antimicrobials [54]. Therefore, assessing the antibiofilm activity of antimicrobial honey is crucial for characterizing it as a protective agent against biofilm-related diseases, such as periodontal disease [55].

Despite its widespread use as a medicinal agent, research on honey’s activity against oral biofilms remains limited. Studies assessing the antimicrobial activity of honey against mature oral biofilms are scarce. One study demonstrated that both Manuka honey and a German multifloral honey inhibited the formation of mature *P. gingivalis* biofilms at a concentration of 10%, reducing bacterial viability within the biofilm for 42 h, but did not completely prevent biofilm formation or eliminate the biofilm [44]. Another study evaluated the effect of supermarket honey from Saudi Arabia on *S. mutans* biofilm formation, finding that 25% and 50% concentrations significantly reduced biofilm growth on polystyrene plates [56]. However, no reports were found on antimicrobial activity against multispecies or subgingival biofilms. Therefore, this research provides novel data that fill a gap in the scientific literature.

This is the first study to investigate the antibiofilm activity of this functional food, providing essential data on its efficacy against a complex multispecies subgingival biofilm composed of 34 species. The use of a multispecies biofilm model, implemented with a Calgary device, adds to the study’s significance, as it closely mimics the in vivo oral environment and presents a greater challenge for antimicrobial testing. Moreover, this research contributes to filling a gap in the literature regarding honeydew honey. We previously employed this model to assess the antibiofilm potential of various bee products, including red propolis, reinforcing its reliability for evaluating natural antimicrobial agents [36].

In our assays, the concentration used to treat the biofilm was based on the minimum inhibitory concentration (MIC) for *P. gingivalis* (2× and 10× MIC). Treatments were administered twice daily for 1 min over a period of four days, with a six-hour interval between applications. Treatment with OHD significantly reduced the overall metabolic activity of the biofilm and showed no statistical difference compared to the positive control (CLX) (*p* > 0.05). Moreover, its strong antimicrobial activity and a lower concentration (2× MIC at 8%) reduced biofilm viability without differing from the gold standard (*p* > 0.05).

Our research group previously studied the antioxidant activity of eight organic honey samples, including OHD, and identified key chemical compounds in the crude honey extracts. The total phenolic content (mg GAE/g) values found in the honey extracts were 73.15 (OH-1), 59.79 (OH-2), 49.79 (OH-3), 52.20 (OH-4), 117.68 (OH-5), 84.08 (OH-6), 83.19 (OHD), and 53.03 (OH-8). Four phenolic compounds were identified: ferulic acid, caffeic acid, rutin, and hesperidin [57]. Additionally, significant amounts of ascorbic acid were found, ranging from 2.75 to 6.22 mg/100 g in OH-3, OH-5, and OHD. Previous studies have demonstrated significant antimicrobial activity for the compounds identified in these organic honey samples [58,59].

Several compounds identified in the OHD sample are known for their anti-inflammatory activity, including apigenin [60], quercetin [61], and hesperidin [62]. Interest in flavonoids has increased in recent years due to their diverse biological activities beyond anti-inflammatory effects, such as antioxidant properties, antidiabetic property, and antimicrobial activity [63].

Two compounds found in the OHD sample belong to the phenolic class known as lignans: anhydrosecoisolariciresinol and matairesinol. These compounds are classified as phytoestrogens and are commonly found in fruits and vegetables. They have also been identified as chemical markers in Brazilian organic propolis type 1. Through intestinal microbiota action, these lignans can be metabolized into enterolactone and enterodiol, and their biological activity is linked to this metabolism [64]. Lignans are reported to have therapeutic potential, including antioxidant, anticancer, gene expression modulation, antidiabetic, estrogenic, antiestrogenic, antimicrobial, and anti-inflammatory properties [65].

Fruits, vegetables, tea, and wine are the main dietary sources of flavonoids for humans. Since bees collect nectar from local flora, the chemical composition of floral and multifloral honey reflects the plant species involved. Consequently, other studies have also identified flavonoids in floral and multifloral honey samples [66].

OHD is a honeydew honey obtained from the Bracatinga tree (*Mimosa scabrella*), differing from floral honey in its origin. While floral honey is produced from flower nectar, honeydew honey is derived from plant secretions or the excretions of plant-sucking insects, as defined by the European Commission Directive (2002) [8]. Compared to floral honey, honeydew honey generally has higher pH, electrical conductivity, liquid absorbance, ash content, and levels of disaccharides, trisaccharides, phenolic compounds, and proteins, which enhance its biological activities. Additionally, honeydew honey is darker in color and has unique sensory characteristics [17,67]. Honeydew honey is known to have a higher concentration of bioactive compounds, such as phenolics, proteins, and amino acids, compared to floral honey. This amplifies its antimicrobial and antioxidant activity, and for this reason, it has attracted the attention of researchers and consumers alike [66,67].

*Honeydew honey* exhibits strong antimicrobial activity, particularly against antibiotic-resistant bacteria. *Heterotrigona itama* honey effectively inhibits *Escherichia coli* and *Staphylococcus aureus*, with bactericidal effects confirmed by endotoxin release assays and scanning electron microscopy, which revealed cellular destruction [28]. Czech *honeydew honey* demonstrated potent antibacterial activity, with a lower minimum inhibitory concentration (MIC) against Gram-positive bacteria than medicinal honeys and comparable efficacy against Gram-negative bacteria [68]. Certified samples of honeydew also exhibited activity against *Klebsiella pneumoniae*, *Streptococcus* spp., *Bacillus cereus*, and *Yersinia enterocolitica*, in addition to serving as a reservoir of bacteria that produce antimicrobial and probiotic metabolites. Its inhibitory effect on urease suggests potential applications in the treatment of specific bacterial infections [6,69].

These findings reinforce *honeydew honey* as a promising natural antimicrobial agent and an alternative in the fight against multidrug-resistant pathogens. Despite the promising antimicrobial activity of honeydew against pathogenic bacteria, its effects on oral microorganisms, particularly periodontopathogens, have not yet been reported. This study provides data that support the sustainable exploration of this functional food, both in the search for bioactive molecules with antimicrobial potential and as a dietary alternative to sugar to help prevent biofilm-associated oral diseases, such as periodontal disease.

A study on the chemical composition of honey and its extracts found a biphasic structure necessary for hydrogen peroxide (H_2_O_2_) production and antibacterial activity [70]. The same research group highlighted that the arrangement of active macromolecules in honey, organized into stable colloidal structures, is crucial for understanding the biological activities influenced by this structural complexity.

In a previous study, we demonstrated the significant anti-inflammatory activity of various organic honeys from the Brazilian Atlantic Forest, which reduced NF-κB activation and TNF-α cytokine levels in vitro. Additionally, OHD reduced NF-κB activation by 87% in an in vitro assay using RAW 264.7 murine macrophages and decreased neutrophil migration by 59% in an in vivo mouse model of carrageenan-induced peritonitis [71]. Since periodontal disease has both microbial and inflammatory components this study aimed to further understand the antimicrobial activity of honey against different species in subgingival biofilms. We conducted a DNA–DNA hybridization assay on in vivo biofilm samples to assess specific reductions based on Socransky’s complexes, analyzing biofilms treated with OHD at 40%, chlorhexidine at 0.12%, and a diluent.

Studies have shown that periodontal disease begins with periodontal dysbiosis. Dysbiosis develops gradually, shifting the relationship between microorganisms and the host to a pathogenic state. The orange complex is first associated with dysbiosis and disease onset, followed by the red complex, indicating a mature biofilm and disease progression [32,72]. *P. gingivalis* adheres to yellow complex pathogens like *S. gordonii*, increasing virulence and causing bone loss [73].

Our DNA–DNA hybridization results showed a significant reduction in 14 species from a 34-species biofilm when treated with OHD at 40% compared to the control (*p* < 0.05). Notably, the biofilm reduction included species from the red complex (*T. forsythia*, *P. gingivalis*, *P. intermedia*, *P. micros*, and *F. nucleatum*) and the orange complex (*S. gordonii*), which are closely linked to periodontal dysbiosis and disease establishment [74,75,76]. The decrease in total bacteria count from the yellow, orange, and red complexes suggests that honey consumption can control these microorganisms’ growth, preventing periodontal disease establishment and progression.

We conducted a periodontal disease model in mice to verify the efficacy of OHD in preventing periodontal disease. Instead of solely applying a ligature silk suture thread to the mice’s first molar, we also exposed the mice to a *P. gingivalis* inoculum (10^7^) for 48 h under anaerobic conditions. For four consecutive days, the mice received 100 µL of *P. gingivalis*. Another study demonstrated that combining *P. gingivalis* with the ligature silk suture thread in the periodontal model exacerbated bone resorption dependent on the RANKL protein [77].

Daily treatment with OHD at 40% five times a day decreased bone loss by 34% compared to the untreated group (Figure 2A). However, the group that received raw honey did not show a statistical difference compared to the untreated group. The treatment frequency was based on a balanced diet of three regular meals and two light meals, totaling five honey feedings a day. A representative image of each experimental group (Figure 2A) showed the preservation of the alveolar bone crest in the furcation area. Damage in that area indicates disease progression, and without regular periodontal treatment, there is an increased risk of molar loss involving the furcation area [78].

A double-blind dental caries score was determined after the periodontal assay by a calibrated examiner, as previously described [39]. None of the mice showed caries lesions or white spots. These findings suggest that despite honey being rich in carbohydrates, rational feeding and short-term use are safe. To verify the antimicrobial potential of OHD in vivo, a ligature silk suture thread was collected for DNA–DNA hybridization. As shown in Figure 2D, the group treated with 40% OHD had a significant decrease in *S. gordonii*, *S. sanguinis*, *F. nucleatum*, *P. gingivalis*, and *Prevotella melaninogenica*, confirming the in vitro results. In the group treated with natural OHD, only *P. gingivalis* showed a significant reduction compared to the control.

Several studies have linked *P. gingivalis*’s presence and virulence to increased bone loss in periodontal disease [79,80]. Therefore, the reduced bone loss in the OHD group may be due to the decreased viability of the *P. gingivalis* biofilm and other periodontal pathogens, as well as its previously evaluated anti-inflammatory activity [71]. These microorganisms stimulate the host immune system to release inflammatory mediators, which in turn stimulate osteoclast activity [81]. Our results suggest that the combined antimicrobial and anti-inflammatory activity of OHD prevented excessive bone loss in the in vivo mouse model.

Surprisingly, OHD at 40% was more effective than raw honey. This enhanced effect may result from both the production of H_2_O_2_ by the glucose oxidase enzyme and the honey’s viscosity. The formation of H_2_O_2_ depends on the honey dilution, as the enzyme is inactive in raw honey [82], and its activation varies with dilution rate [83]. Previous studies have shown a strong correlation between H_2_O_2_ levels and bacterial growth inhibition by honey [82].

Even at high dilutions, H_2_O_2_ remains in the solution and inhibits microbial viability to varying degrees [84]. We hypothesize that raw honey does not activate its glucose oxidase enzyme sufficiently to produce antimicrobial quantities of H_2_O_2_. Additionally, the viscosity of honey may hinder biological compounds from effectively reaching the ligature region. Another factor is the mice’s self-cleaning behavior, which can prevent honey from reaching the ligature area. In contrast, mice treated with 40% OHD, which had active glucose oxidase producing H_2_O_2_ and a fluid consistency to reach the ligature, did not exhibit a self-cleaning behavior.

No other studies in the scientific literature have evaluated the biological activity of honey specifically in an in vivo model of periodontal disease. In our study, honey treatment began on the fourth day after the installation of a ligature silk suture thread, when bone resorption began [37]. Considering that honey is a food, not a medicine, OHD could potentially reduce the risk of periodontal diseases if included rationally in the diet of healthy individuals.

## 5. Conclusions

Our findings demonstrated that OHD, a Brazilian organic honey from the Atlantic Forest, exhibited significant antimicrobial activity both in vitro and in vivo, particularly against periodontal pathogens, while also reducing bone loss. The higher phenolic content and unique composition of OHD likely contribute to its potent antimicrobial and anti-inflammatory effects. Given these results, the responsible consumption of this honey variety may serve as a potential adjunct in the prevention and/or treatment of biofilm-associated oral diseases.

## Figures and Tables

**Table 1:** Antimicrobial activity (MIC and CBM) from eight Brazilian organic honey samples against *P. gingivalis* W83.

Honey Samples and Internal Control	*P. gingivalis* W83
	MIC (%/µg/mL)|MBC (%/µg/mL)
OHD	4.0/40,000|6.0/60,000
Metronidazole	0.0000039/0.039|0.0000078/0.078

**Table 2:** Phytochemical profile by LC-ESI-IT-MS/MS of organic honey- type 7.

Compound	RT (min)	*m*/*z*	Fragmentation	Molecular Formula
Apigenidin isomer (I)	5.5	256.07	(MS2) **256.05**; 255.00; 257.05; 253.8	C_15_H_11_O_4_
Matairesinol	6.4	359.14	(MS2) **341.14**; 323.08; 274.97; 342.12	C_20_H_22_O_6_
Quercetin	12.7	303.05	(MS2) **284.98**; 285.93; 303.03; 301.06	C_15_H_10_O_7_
Apigenidin isomer (II)	18.2	256.07	(MS2) **256.04**; 254.94; 257.04; 238.88	C_15_H_11_O_4_
Hesperidin	24.2	611.18	(MS2) **302.87**; 465.08; 303.89; 575.22	C_27_H_30_O_16_
5,6,7,3′,4′ pentahydroxyisoflavone	13.5	303.02	(MS2) **284.97**; 285.94; 303.04; 257.81	C_15_H_10_O_7_
Anhydrosecoisolariciresinol	25.1	345.16	(MS2) **327.09**; 200.78; 164.73; 136.76	C_20_H_24_O_5_

Bold values are the main precursor ion in the fragmentation. Rt: retention time in minutes. Roman numerals indicate isomers of the same compound.

## Data Availability

The original contributions presented in this study are included in the article/Supplementary Materials. Further inquiries can be directed to the corresponding author.

## Supplementary Materials

The following supporting information can be downloaded at: https://www.mdpi.com/article/10.3390/foods14060997/s1, Table S1: Microorganisms used in the formation of subgingival biofilm.
