# Supplementary material for: Brazilian Organic Honeydew Reduces In Vitro and In Vivo Periodontal Disease-Related Subgingival Biofilm
Source: Foods. 2025 Mar 14;14(6):997. doi: 10.3390/foods14060997 (PMC11941748; doi:10.3390/foods14060997)
Supplement: Supplementary file 1 [file foods-14-00997-s001.zip › foods-3488278-supplementary.pdf]

Table S1: Microorganisms used in the formation of subgingival biofilm.

Especies	ATCC code
Actinomyces gerencseriae	ATCC 23840
Actinomyces israelii	ATCC 12102
Actinomyces naeslundii	ATCC 12104
Actinomyces oris	ATCC 43146
Actinomyces odontolyticus	ATCC 17929
Veillonella parvula	ATCC 10790
Streptococcus gordonii	ATCC 10558
Streptococcus intermedius	ATCC 27335
Streptococcus mitis	ATCC 49456
Streptococcus oralis	ATCC 35037
Streptococcus sanguinis	ATCC 10556
Streptococcus anginosus	ATCC 33397
Streptococcus mutans	ATCC 25175
Aggregatibacter actinomycetemcomitans	ATCC 29523
Capnocytophaga gingivalis	ATCC 33624
Capnocytophaga ochracea	ATCC 33596
Capnocytophaga sputigena	ATCC 33612
Eikenella corrodens	ATCC 23834
Campylobacter concisus	ATCC 33237
Campylobacter gracilis	ATCC 33236
Campylobacter rectus	ATCC 33238
Campylobacter showae	ATCC 51146
Eubacterium nodatum	ATCC 33099
Eubacterium saburreum	ATCC 33271
Fusobacterium nucleatum subsp. nucleatum	ATCC 25586
Fusobacterium nucleatum subsp. polymorphum	ATCC 10953
Fusobacterium nucleatum subsp. vincentii	ATCC 49256
Fusobacterium periodonticum	ATCC 33693
Parvimonas micra	ATCC 33270
Prevotella intermedia	ATCC 25611
Prevotella nigrescens	ATCC 33563
Prevotella melaninogenica	ATCC 25845
Streptococcus constellatus	ATCC 27823
Tannerella forsythia	ATCC 43037
Porphyromonas gingivalis	ATCC 33277
Gemella morbillorum	ATCC 27824
Leptotrichia buccalis	ATCC 14201
Neisseria mucosa	ATCC 19696
